# Presence of an HO-1 expression threshold in renal glomeruli

**DOI:** 10.1016/j.dib.2015.11.001

**Published:** 2015-11-10

**Authors:** Maria G Detsika, Vassileios Atsaves, Apostolos Papalois, Elias A. Lianos

**Affiliations:** a 1st Department of Critical Care Medicine & Pulmonary Services, National and Kapodistrian University of Athens School of Medicine, Athens, Greece; b Research and Experimental center of ELPEN Pharmaceuticals, Athens, Greece; c Division of Nephrology, Department of Medicine, Robert Wood Johnson Medical School, Rutgers Biomedical and Health Sciences, New Brunswick, NJ, United States

**Keywords:** Glomeruli, Heme oxygenase, Kidney

## Abstract

This article reports data describing HO-1 expression patterns of heme oxygenase (HO)-1 in isolated rat glomeruli and in cultured glomerular epithelial cells (GEC) in response to its natural substrate heme. Qualitative and quantitative data are presented to support presence of a HO-1 expression threshold in glomeruli but not in GEC. Interpretation of our data and further insight into HO-1 expression pattern in glomeruli may be found in ‘HO-1 expression control in the rat glomerulus’ [1].

**Specifications Table**

**Table:** 

Subject area	*Biology*
More specific subject area	*Heme oxygenase (HO)-1, cytoprotection, kidney, glomerulus*
Type of data	*Text file and figures*
How data was acquired	*Western blotting, Real-time PCR amplification*
Data format	*Analysed*
Experimental factors	*Glomeruli isolated from kidneys or glomerular epithelial cells (GEC) were incubated with various concentrations of hemin or volumes of hemopexin (HPX) deficient serum for 18 h*
Experimental features	*Pre-treated glomeruli or GEC were processed for western blotting or Real-time PCR amplification*
Data source location	*Athens, Greece*
Data accessibility	*Data are supplied with this article*

**Value of the data**

• These data support presence of an HO-1 expression threshold in the renal glomerulus in response to its natural substrate, heme.
• This threshold may explain the poor response of the renal glomerular microvasculature to HO-1 inducers in contrast to renal tubules.
• Absence of this threshold in one of the key cellular components of the glomerulus (GEC) demonstrates that HO-1 expression control occurs only when the glomerulus preserves its integrated tricellular structure and highlights the importance of studying glomeruli as such.

**Data**

The data presented in this article further characterize the expression pattern of HO-1 in the rat glomerulus and highlight differences of this expression pattern compared to the expression pattern of a key cellular component, glomerular epithelial cells (GEC). Although, HO-1 has an established protective role in the kidney in which it was shown to minimize injury, a marked induction can be detrimental. In this context, we previously demonstrated that HO-1 induction in the renal glomerulus in response to its natural substrate/inducer, heme, reaches a threshold beyond which protein synthesis is halted and HO-1 protein levels are markedly reduced. The present data demonstrate that this threshold also occurs at the HO-1 mRNA level (Fig. 1a) and is not dependent on release of iron (Fig. 1b), one of the heme:HO reaction byproducts known to be cytotoxic when HO-1 activity reaches high levels. The threshold is also independent of the metal (Fe^++^) moiety of heme (Fe^++^Protoporhyrin) as it was also observed when glomeruli were exposed to Cobalt (Co) protoporphyrin, as described in the article to which these data relate [1]. The data presented also demonstrate that the heme-mediated HO-1 induction threshold is inversely proportional to availability of heme. Specifically, increasing availability of free heme by incubating glomeruli with serum lacking hemopexin (HPX), a heme scavenger, lowers the HO-1 induction threshold (Fig. 2a and b). Finally, the data demonstrate that, in contrast to isolated glomeruli, heme-mediated HO-1 induction threshold is not reached in a cellular component of glomeruli (GEC) (Fig. 3).

## 1 Reagents

Anti-HO-1 antibody was purchased from Assay Design and R&D systems respectively. Anti-β-actin antibody, heme (hemin) and desferoxamine (DFO), were obtained from Sigma-Aldrich. Hemopexin deficient (HPX^−^) serum was a kind gift from Dr. EmanualaTolosano, Molecular Biotechnology Center, University of Torino, Italy.

## 2 Rats

Adult male Sprague-Dawley rats, 300 g in body weight, were employed in this study. Animals were reared in accordance to the European Union Directive for care and use of laboratory animals and all procedures were approved by the Hellenic Veterinary Administration and the ethical committee of ‘Evangelismos’ Hospital.

## 3 Isolation and treatment of glomeruli

Glomeruli were isolated from kidneys of wild type (WT) rats by an established differential sieving method [2] and incubated at 37 ˚C in a 5% CO_2_ environment in Dulbecco’s modified Eagle׳s medium (DMEM) containing 10% complete (HPX^+^) serum or defined amounts (*v*/*v*) of HPX^−^serum. Glomeruli were incubated with defined concentrations of hemin dissolved in dimethylsulfoxide (DMSO) in the presence of HPX^+^ or HPX^−^serum. Negative control samples consisted of glomeruli incubated with vehicle (DMSO) only. Protein extracts were prepared using lysis buffer (150 mM NaCl, 50 mM Tris pH 8.0, and 1% Triton X containing a protease inhibitors cocktail) and concentration was determined by the Bradford assay. RNA was extracted by an established Trizol-based method.

## 4 Cell culture

Primary rat GEC were a kind gift of Dr. B.S. Kasinath, Nephrology Division, (University of Texas at San Antonio). Cells were routinely cultured in Dulbecco׳s Modified Eagle׳s Medium (DMEM) containing 10% Fetal Bovine serum (FBS) in a humidified incubator with 95% air and 5% CO_2_.

## 5 Western blotting

Protein lysates were resolved by sodium dodecylsulphate-polyacrylamide electrophoresis (SDS-PAGE), transferred onto polyvinyledinedifluoride (PVDF) membrane and probed with primary antibodies overnight. Horseradish peroxidase conjugated secondary antibodies were used for detection and a chemiluminescence substrate was used for visualization.

## 6 Reverse transcription reaction and Real-time PCR amplification

Glomerular RNA concentration was determined by spectrophotometry. Reverse transcription reactions were performed using TaqMan Reverse Transcription Reagents kit (Applied Biosystems). Real-time PCR was carried out at the following conditions: 25 °C for 10 min, 48 °C for 30 min and 95 °C for 5 min. Each reaction consisted of 2 μl primer-probe assay mix (IDT), 10 μl Master Mix (Applied Biosystems) and 8 μl cDNA. Values were analysed by the ΔΔCT method.

## 7 Statistical Analyses

Values are presented as mean±SE (standard error). Statistical analyses were performed with either *t*-test, where applicable, or analysis of variance (ANOVA) for more than two group comparisons. When significant, post hoc analysis was performed, with the least significant difference (LSD) test. A *p* value<0.05 was chosen as statistically significant.

## Figures and Tables

**Fig. 1:**
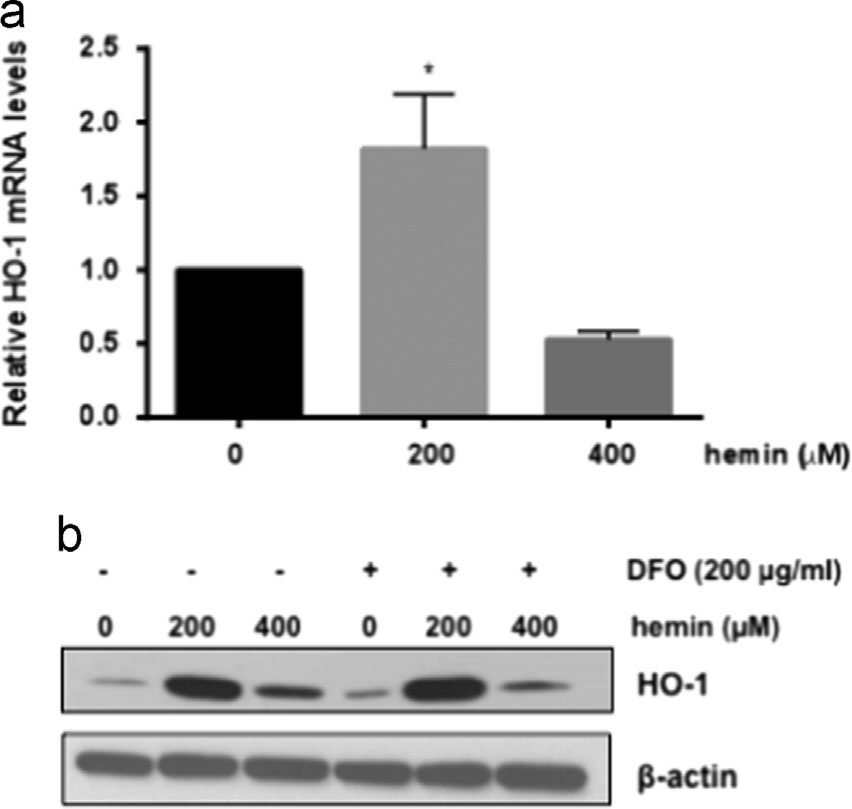
Glomerular HO-1 threshold is independent of Fe. Glomeruli were isolated and treated with increasing concentrations of hemin (200–400 μM) for 18 h. (a) mRNA levels were determined by Real-time PCR amplification. Mean±SEM values from three independent experiments *p<0.05. (b) Glomeruli were isolated and treated with increasing concentrations of hemin (200–400 μM) for 18 h in the presence or absence of a Fe chelator, desferoxamine (DFO). Total protein lysates were analysed by western blotting for HO-1 protein. Representative western blot from three independent experiments.

**Fig. 2:**
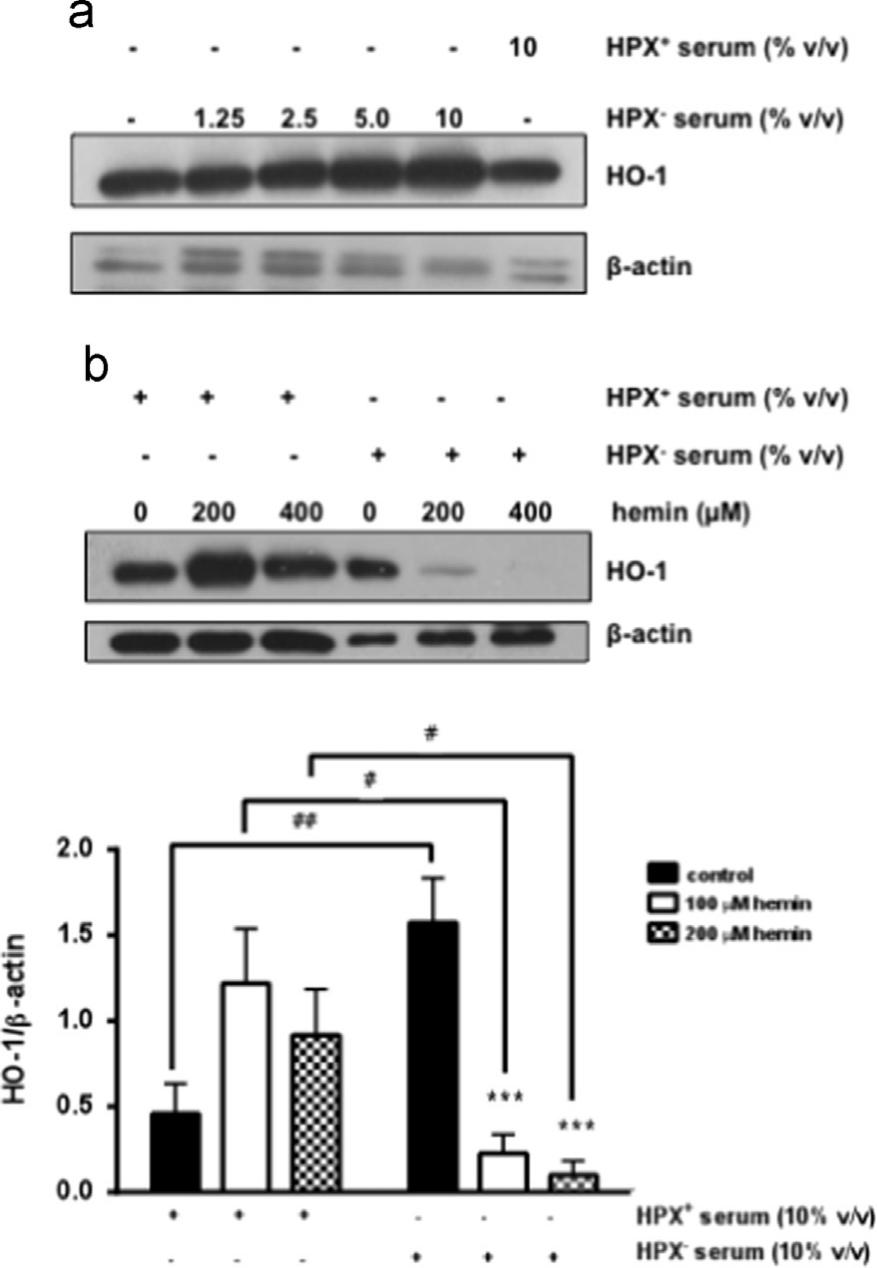
Glomerular HO-1 threshold is dependent on availability of heme. (a) Glomeruli were isolated and incubated with media containing no serum, various amounts of HPX^−^ serum (*v*/*v*) and HPX^+^ serum (10%) for 18 h. Total protein lysates were analysed by western blotting for HO-1 protein. Representative western blot from three independent experiments. (b) Glomeruli were isolated and treated with increasing concentrations of hemin (200, 400 μM) in the presence of 10% HPX^+^ or 10% HPX^−^ serum for 18 h. Total protein lysates were analyzed by western blotting for HO-1 protein. Representative western blot from three independent experiments. Mean±SEM from densitometry values are presented in the graph. #*p*<0.05, ##*p*<0.01, ***p<0.001 compared to no hemin in the presence of HPX^−^ serum.

**Fig. 3:**
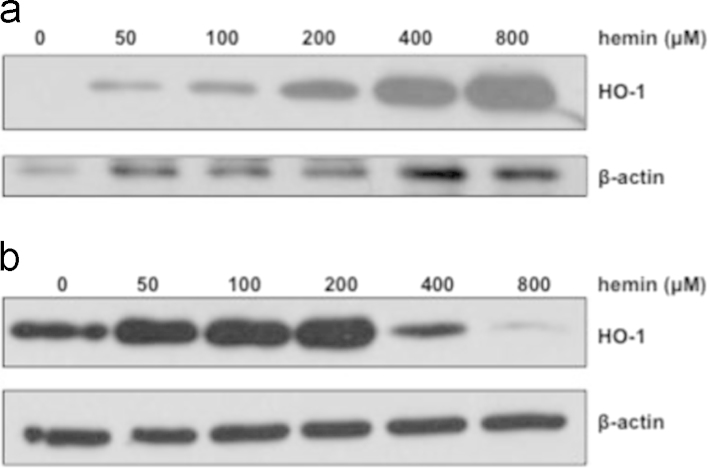
Absence of HO-1 threshold in glomerular epithelial cells (GEC). (a) GEC were treated in the presence of increasing concentrations of hemin (50–800 μM) for 18 h. Total protein lysates were analysed by western blotting for HO-1 protein. Representative western blot from three independent experiments. (b) Glomeruli were incubated with increasing concentrations of hemin (50–800 μM) for 18 h. Total protein lysates were analysed by western blotting for HO-1 protein. Representative western blot from three independent experiments.
